# Understanding the capacity development of faculty development programs: a sequential explanatory mixed methods study

**DOI:** 10.1186/s12909-024-05715-5

**Published:** 2024-07-10

**Authors:** Mahla Salajegheh, John Sandars, Azim Mirzazadeh, Roghayeh Gandomkar

**Affiliations:** 1 Department of Medical Education, Medical Education Development Center, Kerman University of Medical Sciences, Kerman, Iran; 2 Edge Hill University Medical School, Ormskirk, UK; 3 Department of Internal Medicine, School of Medicine, Tehran University of Medical Sciences, Tehran, Iran; 4 Health Professions Education Research Center, Tehran University of Medical Sciences, Tehran, Iran; 5 Department of Medical Education, School of Medicine, Tehran University of Medical Sciences, No. 57, Hojatdoust St., Keshavarz Blvd, Tehran, 1416633591 Iran

**Keywords:** Faculty development, Capacity development, Organizational change, Mixed methods, Medical education

## Abstract

**Background:**

Faculty development programs can bring about both individual and organizational capacity development by enhancing individuals’ attitudes, values, and skillsto enable them to implement organizational change. Understanding how faculty development programs produce capacity development, and the influencing factors, requires further understanding. This study aimed to explore the perceptions of the participants of a faculty development program about the capacity development features of the program and the influencing factors.

**Methods:**

A sequential explanatory mixed methods design was used. Faculty members were surveyed about their perspectives on capacity development of faculty development. Subsequently, 22 interviews were conducted with the respondents to deepem understanding of the survey results. Interview transcripts underwent conventional content analysis.

**Results:**

A total of 203 completed the questionnaire. Most of the faculty highly agreed that the faculty development programs had produced capacity development. The combined data identified (a) “quality of faculty development programs”, underscoring the significance of robust and comprehensive initiatives, (b) “development in instruction”, emphasizing the importance of continuous improvement in pedagogical approaches (c) “development in professionalism”, highlighting the necessity for cultivating a culture of professionalism among faculty members, (d) “development in attitude towards education”, emphasizing the role of mindset in fostering effective teaching practices, and (e) “supporting faculty development programs”, with fostering organizational growth and innovation. Important barriers and facilitators of the capacity development process included several organizational, interpersonal, and individual factors.

**Conclusion:**

The study identified specific features of the capacity development process in the context of a faculty development program and highlighted the importance of these programs in producing changes in both individuals and within the wider organizational system. Several factors that enabled and constrained the capacity development process were also identified. The findings of the study can inform future implementation of faculty development programs for capacity development.

**Supplementary Information:**

The online version contains supplementary material available at 10.1186/s12909-024-05715-5.

## Introduction

Over recent years, the emphasis of faculty development programs in health professions education has shifted from the preparation of faculty members for teaching to facilitating capacity development for organizational change [1]. Capacity development of faculty development programs is a process of strengthening individuals’ attitudes, values, behaviors, and abilities [2]. This process also enables the teacher to increase their participation within the wider system, with subsequent improvement of the collective ability of organizations to support change and progress [3, 4]. An important aspect of this process is that it enhances the collective abilities and relationships of individuals within a wider system to foster a sense of ownership and development in the organization, which supports a new level of performance [5].

Some previous studies have highlighted the contribution of faculty development programs to individual and collective capacity development in medical education. Frantz et al. in two consecutive studies investigated the contribution of a faculty development program to individual and collective capacity development in sub-Saharan Africa [2, 6]. Further research by Salajegheh et al. has identified the capacity development indicators of faculty development programs that contributed to organizational development [7]. Kolomitro et al. explored the organizational factors as valued contributors to the educational mission that affect the capacity of faculty developers [1]. However, there has been little understanding of how capacity development has been produced by faculty development programs [8–10]. Most previous research on faculty development programs has had a particular lens for capacity development, which is the human resource training lens with a limited focus on individual teaching knowledge and skills, and increasingly professional identity development. However, considering the pace of health system transformations, funding concerns, and innovation in caring for patients, there is an urgent need for faculty development programs to produce organizational change globally [11–13].

Effective organizational change needs an alternative lens for capacity development. Capacity development theory (Morgan, 2006) provides a lens to deepen our understanding of capacity development for faculty development programs. Based on this theory, the critical characteristics of capacity development are product, permanence, processes, and contextual factors. The product refers to what individual and organizational capacities have been developed. The permanence returns to the capacities that are applied during or after the program to ensure the long term success of the program. The processes refer to how both the product and permanence have been developed. The contextual factors address what enables and constrains the processes [14]. Morgan’s capacity development theory has been used previously in different settings outside of medical education. For instance, researchers have studied short and long-term education programs for capacity building for climate change adaptation [15]. This study revealed that rigorous coordination and monitoring of training efforts and appropriate institutional support were essential to enhance organizational development. Sheikhattari et al. have also described how they used capacity development theory to effectively establish a community-campus network of research partnerships [16]. While Morgan’s capacity development theory has been applied in various contexts outside of medical education, there has been a lack of its use to investigate the nature of capacity development within faculty development programs.

The aim of the study is to to explore the perceptions of the participants of a faculty development program about the capacity development of thefaculty development program at Tehran University of Medical Sceinces (TUMS) and the influencing factors. By revealing the complexities of faculty developmentprograms, the findings of this research will inform policymakers’ decisions for future planning and consider the best possible resources to reinforce or modify the subsequent programs.

Since 2003, Education Development Centre of TUMS has implemented a longitudinal educational faculty development program called “Basic Teaching Skills Course”. This program aims to address the needs of health professional teachers to accomplish their educational roles and to pursue the mission of organizational excellence at TUMS. The “Basic Teaching Skills Course” intends to deliver the basic subjects of educational effectiveness, such as instructional design, teaching methods, and student assessment. Its main focus is on new or less experienced faculty members, and it is also compulsory for all faculty members who wish to be promoted to associate professor rank. This longitudinal program lasts for 48 h in total and has been implemented in both in-person and virtual (synchronous and asynchronous) learning formats. The strategies of instruction consisted of small-group discussions, interactive lectures, as well as assignments and feedback. Faculty members from all eleven schools of TUMS participate in this program.

## Methods

### Setting

This study took place from April 2019 to March 2020 at Tehran University of Medical Sciences.

### Participants

All faculty members who participated in the “Basic Teaching Skills course” between 2014 and 2017, were invited to participate in this study.

### Study design

We employed an explanatory sequential mixed methods study design that comprised administering a survey followed by conducting semi-structured interviews. We designed the research sequentially to first use a quantitative survey to collect the participants’ perspectives on capacity development for faculty development and second conducting semi-structured interviews to provide an extra explanation and a deeper understanding of the complex phenomenon of the capacity development process [17]. Based on the capacity development theory assumptions, mixed methods design can elucidate the complex concept of capacity development [14].

The Ethical Review Board of the National Agency for Strategic Research in Medical Education approved the study (No. 970,080).

### Quantitative component

#### Participants and procedure

We collected the participants’ perspectives on capacity development for faculty development in the context of the “Basic Teaching Skills Course” using the previously validated capacity development questionnaire for faculty development (CDQ-FD) [18, 19]. The questionnaire consists of 21 five-point Likert-type scales from 1 (very low) to 5 (very much) across three domains: development and innovation in the teaching and learning process (items 1–13), development and sustaining faculty development programs (items 14–17) and development of educational leadership and management (items 18–21). The maximum score of the CDQ-FD is 105 (Domain 1 = 65, Domain 2 = 20, and Domain 3 = 20) and the minimum score is 21 (Domain 1 = 13, Domain 2 = 4, and Domain 3 = 4).

The CDQ-FD was sent by email to 311 faculty members who had participated in a “Basic Teaching Skills course” between 2015 and 2018. The non-responders to the questionnaire were followed up at four weeks using email and social media.

#### Data analysis

Data were processed and analyzed using version 24.0 of SPSS (SPSS Inc., Chicago). Descriptive statistics, including numbers, frequencies, mean, standard deviation (SD), and total score distributions were computed. We considered the “very much”, “much” and “average” scale categories by presenting the frequencis at the questionnaire item level. Differences in the responses between schools and scores of the questionnaire were assessed using analysis of variance (ANOVA) with LSDʼs post-hoc analysis. An Independent T-test was used to assess differences in the responses between two groups, such as gender (Male, Female), educational department (Clinical science, Basic science), rank of faculty (Assistant professor, Associate professor), and the experience of being faculty member (1–5 years experience, 6–10 years experience). A two-tailed *p*-value of less than 0.05 was considered statistically significant.

### Qualitative component

#### Data collection

Face-to-face, semi-structured interviews using an interview guide (Appendix 1) were performed to explore the breadth and depth of capacity development and the influencing factors. The interview guide was drafted by the first author, with a list of open-ended questions to explore the desired concepts. Then the research team arranged the questions in a logical sequence to ensure a smooth flow of the interview and to facilitate a natural conversation. Also, they included probe questions that can help to clarify any ambiguities, and to explore different perspectives.

Participants were selected using two types of purposive sampling from the survey respondents. We used extreme case sampling, focusing on samples that were on two deviants of the respondent’s spectrum (individuals with high or low scores in the questionnaire) [20]. This technique of purposive sampling is optimal because these two groups of respondents are information-rich and can be valuable illustrative of the experiences, attitudes, and perceptions of the pros and cons of capacity development for the faculty development programs. The second type of sampling was selecting typical cases [21] from those who were in the mean of the respondent’s spectrum to provide the ability to compare the findings. Data collection and analysis were performed concurrently from May 2019 to February 2020. One author (M.S.) conducted all interviews. The interviewer actively participated in reflexivity and positionality exercises before the interviews by reflecting on her values and beliefs with another member of the research team (R.G.). These exercises were pivotal in promoting self-awareness, transparency, and ethical conduct throughout the interview process. Another member of the research team (R.G.) reviewed transcripts after each interview for the first 5 ones and provided feedback to the interviewer about areas for further probing as the expert check process. Interview questions were also modified during these meeting discussions. The interview questions were directed by survey results and capacity development theory resulting in more authentically grounded results. Data collection was continued until saturation was achieved. All interviews were audio-recorded with permission of participants and transcribed verbatim at the earliest possible opportunity. We gave each participant a number to report qualitative data.

#### Data analysis

The data gathering and analysis was conducted iteratively. The analysis strategy was conventional content analysis and consisted of three steps. Firstly, one author (M.S.) reviewed each transcript several times to ensure that it conveyed the viewpoints of participants and then coded the transcripts after each interview to inductively construct a preliminary set of descriptive codes as a code book. In step two, constant comparative analyses by M.S. and R.G. enabled the exploration of overarching subcategories and categories. In the third step, the emerging results were shared with the full research team. The team members shared their individual perspectives, interpretations, and insights derived from the data analysis and engaged in extensive discussions and reflections until agreement on categories and subcategories was reached. In all of this process, the research members had regular team meetings to facilitate co-construction of findings and resolve differences in interpretation of data and careful documentation of decisions made throughout the study design, data collection, and analysis to establish confirmability and dependability. Finally, we transformed the categories into the capacity development characteristics: product, permanence, processes, and contextual factors.

#### Rigor and trustworthiness

The credibility of data was confirmed through prolonged engagement, peer debrief and purposeful sampling. To confirm confirmability, the extracted codes and categories were agreed upon by the research team. A thick description of context was employed to enhance the transferability of data. To confirm dependability, and the process and product of the several randomly selected analyzed transcripts was presented to a qualitative researcher outside of the team and the plausibility of the findings confirmed that the analyses and interpretations were justifiable [22].

## Results

### Quantitative phase

From 311 questionnaires, 203 were returned with a response rate of 64.9%. Female participants (49.3%) were almost equal in number to the male participants. Most of the participants were affiliated with clinical science departments (71.2%). Most participants (88.8%) were assistant professors, and 10.2% were associate professors. Most (71.7%) had 1–5 years of experience being a faculty member and 69.8% were from the school of medicine.

### Descriptive findings

Most participants indicated the capacity development of faculty development programs in all items above average. The score of the “Development and sustaining faculty development programs” domain (*M* = 3.45, SD = 0.94) was higher than “Development and innovation in teaching and learning process” (*M* = 3.32, SD = 0.76) and “Development of educational leadership and management” (*M* = 3.31, SD = 0.86).

#### Domain 1. Development and innovation in teaching and learning process

**Fig. 1:**
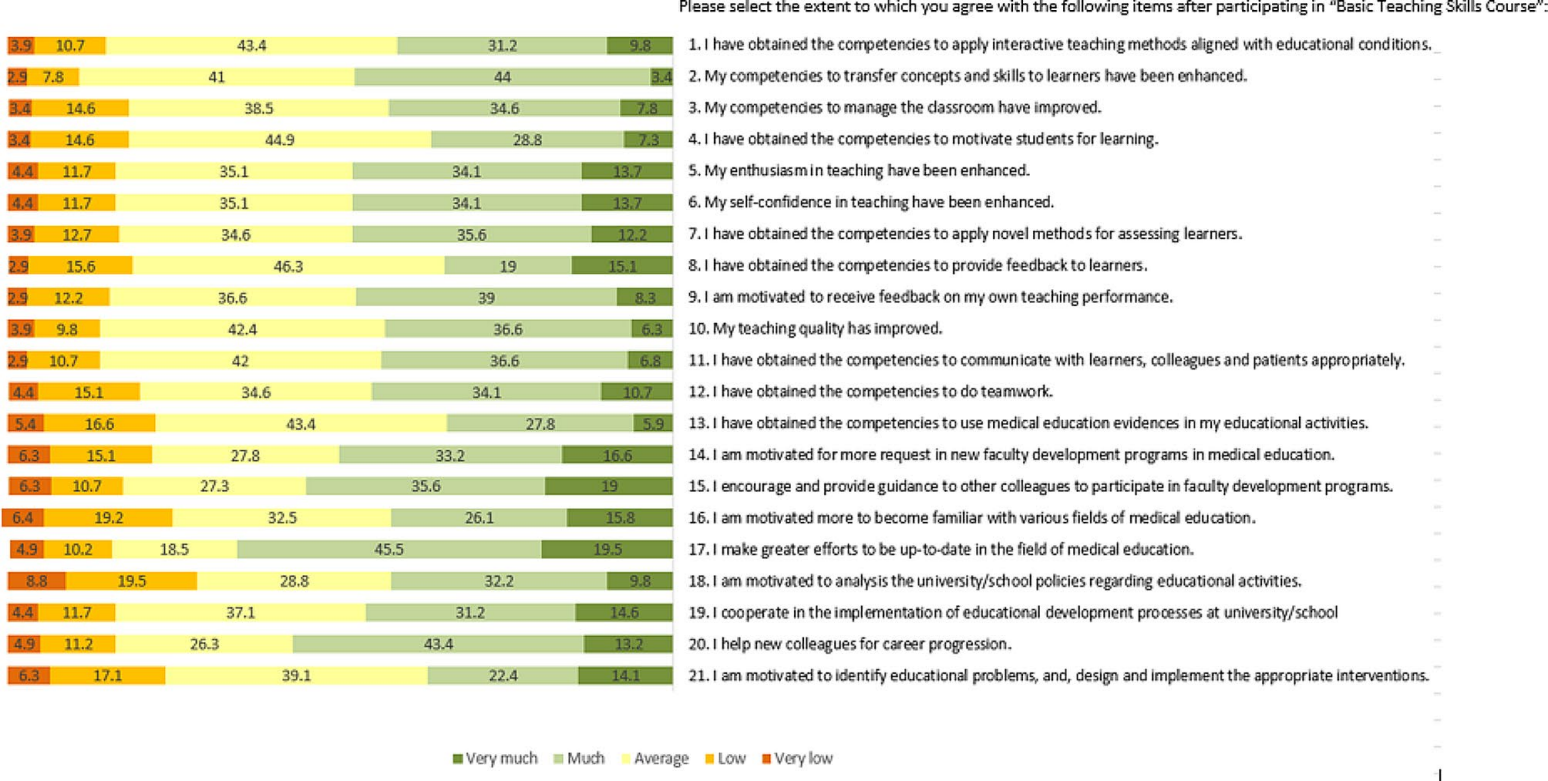
Frequency of responses to items in CDQ-FD

The highest capacity development indicator was “My competencies to transfer concepts and skills to learners have been enhanced.” (88.4%). The lowest score belonged to “I have obtained the competencies to use medical education evidence in my educational activities” (77.1%) (Fig. 1).

#### Domain 2. Development and sustaining faculty development programs

Most of the responders gave the highest scores to “I make greater efforts to be up-to-date in the field of medical education” (83.5%). In comparison “I am motivated more to become familiar with various fields of medical education” (74.4%) gained the lowest score (Fig. 1).

#### Domain 3. Development of educational leadership and management

The highest scores were belong to “I cooperate in the implementation of educational development processes at university/school” and “I help new colleagues for career progression” (82.9%). The lowest scores were “I am motivated to identify educational problems, and design and implement the appropriate interventions” (70.8%) (Fig. 1).

### Analytical findings

We looked for associations between the demographic data and the three domains of the questionnaire. There were no significant differences across the scores of the three domains when compared by male (*M* = 71.25, SD = 16.02) and female (*M* = 26.26, SD = 15.11), (*p* = 0.365, t = 0.90). There was a statistically significant association in the scores of “development and innovation in teaching and learning process”, “development of educational leadership and management” and the total score of the CDQ-FD questionnaire when comparing by the educational department. The score of clinical science faculty (*M* = 71.93, SD = 14.31) was significantly higher than basic science faculty (*M* = 65.48, SD = 18.10), (*p* = 0.009, t = 2.62). The scores of all domains and the total score of the questionnaire were significantly negatively correlated with the rank of the faculty. The assistant professors (*M* = 71.21, SD = 15.28) scored significantly higher than associate professors (*M* = 62.04, SD = 16.00), (*p* = 0.010, t= -2.59). We also repeated the above analysis by comparing the scores of all domains and the total score of the questionnaire with the experience of being a faculty member and this showed that the faculty with more experience had a lower score (*M* = 63.04, SD = 16.09) than faculty with lower experience (*M* = 70.26, SD = 15.57), (*p* = 0.000, t= -0.26). Measures of ANOVA and LSDʼs post-hoc analysis indicated a significant difference between the scores of all domains and the schools (*p* = 0.045, F = 2.065), except for Pharmacy.

### Qualitative phase

Twenty-two faculty consented to and engaged in interviews. Female interviewees (54.5%) were almost equal in number to the male. Most (81.8%) were assistant professors, and the rest were associate professors. Most of the participants were affiliated with clinical science departments (68.2%), and 40.9% were from the school of medicine. Half of the participants had 1–5 years experience of as a faculty member.

After analysis of 22 semi-structured interviews, six categories were identified related to the capacity of faculty development programs. These categories were compatible with the critical characteristics of capacity development of Morgan’s capacity development theory (Table 1).

**Table 1:** Identified categories and subcategories related to capacity development for faculty development programs

Critical characteristics of capacity development based on Morgan’s capacity development theory	Category	Subcategory
Product	Development in attitude toward education	Understanding the importance of education
		Gaining a perspective on the dynamism of education
		Gaining a holistic perspective on education
	Development in instruction	Instructional planning
		Teaching capabilities
		Student assessment practices
	Development in professionalism	Communication skills
		Professionalism
Permanence	Supporting faculty development programs	Desire to take part in future programs
		Guiding and encouraging peers to participate
Processes	Quality of faculty development programs	Instructional content
		Instructional method
		Program management
		Program follow up
Contextual factors	Barriers and Facilitators of the capacity development process	Organizational factors
		Interpersonal factors
		Individual factors

#### Development in attitude toward education

The “development in attitude towards education” category was compatible with the “product” in capacity development features. Some faculty talked about their attitudes towards education in terms of understanding the importance of education, gaining a perspective on dynamism of the education, and gaining a holistic perspective on education after participating in the “Basic Teaching Skills Course” such as “Now, I am looking at education such as a science that has different disciplines which are classified and specialized with theoretical and scientific foundations. I used to think that being a teacher is instinctive, but currently, I realize that aside from these innate skills, it can be trainable” (P18).

#### Development in instruction

##### Instructional planning

Some episodes of this category refer to the development of instructional design and planning competencies after participating in faculty development programs. As P12 shared, “Before this, it was like I swam against the currents, but when I have been planning for each session at the beginning of the semester, the flow was completely in the same direction”.

##### Teaching capabilities

One of the other impacts of faculty development interventions on capacity development is presented in the development of teaching capabilities including gaining knowledge, as “I was not familiar previously with different teaching methods” (P2), and applying innovative and suitable teaching methods as “My colleagues who had participated in the “Basic Teaching Skills Course” and I am using flipped-classroom and small group technique” (P13) or “Some of faculty have been implementing the flipped-classroom method and problem-based learning and report on it” (P15).

##### Student assessment practices

Also, some participants reported how their student assessment capacities had changed positively such as “We organized an examination committee in our department which aims to revise the questions and try to correct their weaknesses” (P8).

#### Development in professionalism

##### Communication skills

Participants stated improvement in their communication skills and professional behavior. As shared by P15, “Based on the feedback from our students, the communication between faculty and students has improved”.

##### Professionalism

Also, some aspects of professionalism improved, as noted by some clinical faculty members, “I have been more respectful to the comments and feedback that I received from residents” (P1), and “I tried to attend to my behavior as a role model for the students” (P3), and “I care more about my communication with the obsessive or aggressive patients, especially in the presence of students” (P17).

#### Supporting faculty development programs

Supporting faculty development programs was compatible with the “permanence” characteristics of capacity development.

##### Desire to take part in future programs

Some participants believed that their willingness to take part in future faculty development programs in medical education has increased. As P22 described, “After participating in “Basic Teaching Skills Course”, I enrolled in the virtual MS course of Medical Education”, or as shared by P18 “My colleagues and I requested to implement that (the same) program in our hospital as well”.

##### Guiding and encouraging peers to participate

Many faculty described guiding and encouraging other colleagues to participate in these programs as the capacity development for faculty development programs, which is represented in comments similar to, “I am following other faculty development programs, and encouraging my colleagues to participate in workshops together” (P20).

#### Quality of faculty development programs

Our findings demonstrated the importance of the “processes” components in capacity development through the “quality of faculty development programs” category. Participants defined quality for four elements of the programs including instructional content, instructional method, program management, and program follow-up.

##### Instructional content

Although faculty members were mostly satisfied with the program’s content, a clinical faculty member mentioned that “The skills taught to basic science or clinical professors are almost same and specific needs of faculty members were not addressed” (P3).

##### Instructional method

The interactive teaching methods such as small-group discussions and role-plays, diversity in materials such as videos, and structured opportunities for assignments, groups, and reflections were identified as positive features of the “Basic Teaching Skills course” related to capacity development of faculty development programs. In this regard, P6 noted, “Practice on how to write the stems, stimulus, and options for MCQs helped me to apply item construction principles”.

##### Program management

Many faculty members believed that some of the features of program management such as time scheduling, and holding the course in an intensive period with no attention to the high workload and various responsibilities of the participants may be a challenge for capacity development of faculty development programs. In the words of P4 and P3, it is respectively stated that “It is very difficult to participate in a weekly course especially when your colleagues do not cover you in your patient care responsibility” and “It would have been better if the classes were implemented in the hospitals or schools, it would be easier to access”.

##### Program follow up

Another aspect that was considered to have contributed to the capacity development was the program follow-up. Lack of connections between the participants and the instructors of the course after the program, to answer the raised questions after applying the new methods in the workplace, are the issues brought up by the number of participants. As P1 commented, “I preferred to contact the lecturers after the course to ask my questions and receive feedback.”

#### Barriers and facilitators of the capacity development process

A large part of participants’ statements denoted the influencing factors on the capacity development process. Some of these factors played the role of facilitators, some were identified as barriers to capacity development and other factors played both facilitator and barrier roles. This highlights the importance of contextual factors in the capacity development process, which is one of the important features of the capacity development theory. This category contains three subcategories including organizational factors, interpersonal factors, and individual factors.

##### Organizational factors

Several organizational factors including the variety of expectations from faculty members and the high workload, unavailability of educational facilities, small contribution of education in the promotion of faculty members, and the caveat in the rewards system for education have been considered as barriers to capacity development of faculty development programs from the participants’ perspectives. Many faculty expressed the difficulties they found in coping with performing different responsibilities such as services, administration, and research which mostly take priority of education, “We have many concerns that among them, education seems no longer important. Most times, there is no time for Mini-CEX and such methods” (P17). The lack of some essential educational facilities was another obstacle to capacity development for faculty development programs, “Our small groups practically fail because we implement this method in a class with long tables that when the students want to talk from one end of the table to the other, it becomes crowded” (P9). Participants highlighted the low contribution of education in the process of promotion and tenure such as “Changes in educational performance are not very effective for faculty in comparison with research in the promotion process” (P20) as a constraint factor to the process of capacity development for faculty development programs. Lack of appreciation and encouragement low compensation for education, and time-consuming changes in the education process were recognized as important barriers to capacity development for faculty development programs. As P19 commented “Even little appreciation or acknowledgment is valuable” or “If education had more income, one would pay more attention to it” (P10).

Some participants highlighted the role of student evaluation of teaching quality as an enabler and the positive changes they adopted in their teaching; “The evaluation encouraged me to use what I have learned to increase my score” (P14) or “The students evaluate faculty and we have to improve ourselves” (P21).

##### Interpersonal factors

Interpersonal factors like support from managers could play a dual role as barriers or facilitators for the capacity development process. For instance, several faculty members identified that educational managers like the chair of the department “Do not believe in these new teaching methods” (P12) or “Prevent us from implementing the new methods” (P13) resulting in a decline in the capacity development process. Conversely, other participants acknowledged the educational managers who showed a positive attitude and provided the opportunity to apply the learned skills during the faculty development. One basic science faculty member mentioned, “I wanted to make a map of the tissues and give it to the students. When I raised it in the department meeting, the chair liked it and helped me to make it possible” (P18). Also, P6 noted “Our chair participated in the “Basic Teaching Skills course” with us, and then we organized a committee in our department based on his suggestion to review and correct the exam items”.

Another interpersonal factor alludes to support from colleagues with positive effects on the capacity development process in our context. According to most faculty, receiving positive and constructive feedback from peers on the new educational experiences is a strong enabler for the capacity development of faculty development programs. As P20 commented, “One of the new things in our department is visiting each other’s classrooms, and then discussing our observations and giving feedback to each other”.

Interpersonal factors included support from the learner as well as a dual role. Faculty recognized insufficient effort for learning by some students, especially when using student-centered teaching methods that require cooperation and interaction of students as the most important obstacle to capacity development of faculty development programs. As P13 noted, “When we apply the flipped-classroom method, most of the students don’t study the material”. On the contrary, high motivation in the majority of learners facilitates the application of new educational methods “We can no longer apply those old teaching methods to these students. These are generation Z with different characteristics. I have to change myself” (P14) or “Most of my colleagues do not know how to deal with this generation and must learn” (P2).

##### Individual factors

Several personal factors such as internal motivation and desire to improve educational performance based on the learned lessons in faculty development programs and a sense of responsibility toward learners were identified as reinforcement for the process of capacity development of these programs. As P2 commented, “After the course, I felt that I had to update myself to the new methods” or “The new faculty members themselves would like to see a change in the system” (P4).

## Discussion

The study had the aim of exploring the perceptions of the participants of a faculty development program about the capacity development of the program and the influencing factors. These findings reveal various organizational capacity developments that were derived from the faculty development program. In addition, the qualitative data provided further insights by exploring the barriers and facilitators of the capacity development process.

We were able to summarize our findings through the lens of critical characteristics of Morgan’s capacity development theory. This mapping provides a comprehensive view of key elements contributing to organizational capacity development of faculty development programs. Based on our findings, the capacity development of these programs starts with the quality of the programs (i.e. processes) in terms of instructional content, methods, management, and program follow-up. These results are in line with previous findings that the quality of faculty development programs and their components and requirements are the primary factors in engaging individuals with these programs [5, 23–25]. Improving the quality of programs leads to better results in both permanence (e.g. supporting faculty development programs) and product (e.g. development in attitude towards education, development in instruction, and development in professionalism). Our results confirm that the expected outcomes (development in instructional planning, teaching methods, and assessment practices) of educational faculty development programs can lead to future changes in the performance of an organization [25]. Our participants in both qualitative and quantitative phases reported that positive changes in attitudes toward education included increased enthusiasm, motivation, and confidence in teaching, and a sense of evolution in their perspective on education provided a basis for the capacity development of faculty development programs. This supports the literature demonstrating that positive perceptions are critical and vital for faculty to perform their educational role successfully [26, 27].

Faculty development can also enhance organizational capacity development by sustaining the capacities developed (i.e. permanence characteristic). Most of our faculty showed their desire to take part in future faculty development programs in medical education in line with Cortezano et al. findings [28]. Our participants also encouraged their peers to participate which can develop relationships and networks of colleagues with a common conceptual language in which they can communicate about their teaching experiences [29] and enhance their identity as educators [30, 31]. Supporting faculty development programs by faculty facilitates the development of a community of practice, with implications for the sustainability of change at the system level [28, 32].

While most faculty development programs focus on improving teaching [33], our findings reveal that capacity development can promote organizational change by enhancing a variety of leadership skills, especially guiding and encouraging peers to participate in further development. These skills have far reaching impacts to improving institutional structure and culture, because capacity development formally develops the next generation of health professions education leaders. By promoting a culture of excellence and accountability, educational leaders can inspire faculty members to embrace change, adapt to new teaching and learning methods, and contribute to a dynamic and thriving academic community [34].

Finally, capacity development theory characterizes that contextual factors can promote or undermine capacities derived in terms of product, permanence, and processes from faculty development programs. Our findings revealed that the organizational, interpersonal, and individual factors affect the capacity development of faculty development programs, positively, negatively, or both. Our findings, while confirming previous studies on the contribution of perceived workplace priorities and institutional culture on faculty development efforts [35] and the role of social support and motivators in enhancing training transfer [36], identified student evaluation of teaching and support from learners included in these factors.

Using Morgan’s capacity development theory has increased our understanding of faculty development as an open system in which individual efforts produuce organizational change. We argue that faculty development programs can result in changes at the organizational level if they follow the capacity development process and address its characteristics comprehensively. Bruggen et al. proposed a 4-C Framework (Competence, Context, Community, and Career) to enhance participation in faculty development and asserted that leaving out any of the framework components will undermine the programs’ effectiveness [37]. Steinert has argued that faculty development affects change with the four conditions of change: develops the “desire” to change, produces the “knowledge” of what to do and how to do it, creates a supportive institutional “environment”, and considers “reward” for changing [38].

### Implications and recommendations

The results of this study have various implications fortheory and practice. The findings strengthen the body of knowledge of faculty development, and especially capacity development for faculty development, by explaining the components and different factors, which were aligned to Morgan’s capacity development theory [14]. In practical terms, our findings can inspire faculty developers to focus on the contentof their faculty development program and its teaching approach to ensure that it can enhance the participants’ contributions to their local environment, while simultaneously contributing to organizations outside their local environment [2]. We strongly recommend policymakers and administrators afford a supportive environment in the university at all levels and provide rewards, incentives, recognition, logistics, and organizational support. This gradually addresses a wider community of professionals who can make a meaningful long-term contribution to health professions education and healthcare at the individual and collective levels. We recommend that future studies explore the long-term impact of faculty development programs by measuring the proportion of program alumni who transition into formal leadership roles and also their impact on organizational change.

### Strengths and limitations

One strength is that we used a conceptual framework that was developed specifically for organizational development research. Therefore, with this framework, we could comprehensively and efficiently identify the key factors that influence the capacity development of faculty development programs. Another strength is that this study explored the experiences and perspectives of health professional educators from a non-Western setting. By focusing on the perception of these faculty, it was possible to “give voice” to the participants, which is an important function of qualitative research. Also, the complex and multidimensional nature of the capacity development process which is coming out of the dynamics involving a multifaceted combination of attitudes, resources, strategies, and skills, both tangible and intangible, and it usually deals with complex human activities which cannot be addressed from an exclusively technical perspective, a triangulation of quantitative and qualitative methods is used to provide a deeper understanding of the complex phenomenon of capacity development.

On the other hand, some limitations of this research should be considered. Firstly, it was based on the experience of participants in one program. Therefore, findings may not be generalizable to other contexts. Further research in other settings is recommended to determine whether these findings generalize over one institution. Secondly, we only studied the viewpoints of faculty members, but it seems that it is necessary to know the views of other stakeholders in the medical education system, such as policymakers of faculty development programs, students, and educational leaders to enhance the richness of the data. Thirdly, two of the researchers were involved in designing and teaching in “Basic Teaching Skills Course”, but they did not participate in data gathering, so they did not interfere with or influence participants’ responses.

## Conclusion

The capacity development of faculty development programs facilitates the development of personal and professional capacities. These developments also positively contribute to changes in the wider health profession education system. Upon closer examination, several factors deserve specific attention. These factors include the “development in attitude towards teaching,” “development in instruction,” and “development in professionalism.” Each of these can be viewed as a distinct element contributing to the overall efficacy of faculty development programs. Furthermore, aspects such as the “product” of these programs, the “supporting faculty development programs” for their continuity, the “quality” of these programs regarding their processes, and the consideration of “environmental, individual, and interpersonal factors” as contextual elements all play crucial roles in strategizing for enhancing the capacity development of faculty development programs.

### Electronic supplementary material

Below is the link to the electronic supplementary material.

Supplementary Material 1

## Data Availability

The datasets used and/or analyzed during the current study are available from the corresponding author upon reasonable request.
