# Supplementary material for: Understanding the capacity development of faculty development programs: a sequential explanatory mixed methods study
Source: BMC Med Educ. 2024 Jul 10;24:744. doi: 10.1186/s12909-024-05715-5 (PMC11234744; doi:10.1186/s12909-024-05715-5)
Supplement: Supplementary file 1 — Supplementary Material 1 [file 12909_2024_5715_MOESM1_ESM.docx]

Appendix [1](https://onlinelibrary.wiley.com/doi/full/10.1111/medu.14418#support-information-section). Interview guide

| **Instructions for the interviewer:**  Review the concept of "capacity development" with interviewees before beginning the protocol and recording.   1. For this study, “capacity development” is defined as changes in human behavior, such as the growth of new attitudes, values, knowledge, skills, and relationships with others, which are generated over time within a complex organizational system. | |
| --- | --- |
| **Questions before modification:**   1. My team and I are trying to understand what is your experience of participating in the “Basic Teaching Skills Course”? and what has happened to the educational activities of you or your colleagues that you can attribute to this course. 2. From your lens, describe the changes because of this course to your organization. Share one of your observations or personal experience. 3. Please elaborate on your perceptions of how participating in the “Basic Teaching Skills Course” has produced some changes in your organization. 4. Based on your experience, what are the factors that led to these organizational changes? What obstacles made the organizational results of these courses less? 5. Anything else you want to share about organizational changes because of the “Basic Teaching Skills Course”? | **Questions after modification:**   1. My team and I are trying to understand what is your experience of participating in the “Basic Teaching Skills Course”? and what has happened to the educational activities of you or your colleagues that you can attribute to this course. 2. From your lens, describe the changes because of this course to your organization. By organization we mean, your educational department, hospital, school, or the university as a whole. Share one of your observations or personal experience. 3. Please elaborate on your perceptions of how participating in the “Basic Teaching Skills Course” has produced some changes in your organization (or is not). 4. Based on your experience, what are the factors that led to these organizational changes? What factors can increase these organizational outcomes? What obstacles made the organizational results of these courses less? 5. What do you suggest to overcome these obstacles? 6. Anything else you want to share about organizational changes because of the “Basic Teaching Skills Course”? |
| **Probe for:**  a) facilitators/obstacles/organizational changes after the faculty development program? | |
